# The potential impact of age, gender, body mass index, socioeconomic status and dietary habits on the prevalence of dental caries among Egyptian adults: a cross-sectional study

**DOI:** 10.12688/f1000research.17892.1

**Published:** 2019-03-01

**Authors:** Marwa M.S. Abbass, Nermeen AbuBakr, Israa Ahmed Radwan, Dina Rady, Sara El Moshy, Mohamed Ramadan, Attera Ahmed, Ayoub Al Jawaldeh

**Affiliations:** 1 Oral Biology Department, Faculty of Dentistry, Cairo University, Cairo, Egypt; 2 Specialized Dental Hospital, Armed Forces Medical Complex, Cairo, Egypt; 3 Independent Researcher, Research door, Limerick, County Limerick, Ireland; 4 Nutrition Unit, World Health Organization Office for Eastern Mediterranean Region, Cairo, Egypt

**Keywords:** Caries, Prevalence, Age, Socioeconomic, Dietary, Education, Egyptian, Adults

## Abstract

**Background** **:** Dental caries is a major public health problem and the most widespread chronic disease to affect individuals throughout their lifetime. Little information exists about the prevalence of dental caries among Egyptian adults. Therefore, this study investigated the dental caries experience among Egyptian adults in correlation with different risk factors.

**Methods** **:** A total of 359 Egyptian adults (age range, 18-74 years) were examined over a period of 3 months, starting on the 15 ^th^ of November 2017 until the 13 ^th^ of January 2018. Socio-demographic data, brushing frequency, body mass index (BMI) and eating habits were recorded and collected using a questionnaire. Dental examination was performed using the Decayed, Missing and Filled tooth (DMFT) index.

**Results** **:** In total, 86.63% of participants had dental caries experience. Of the participants, 60.45%, 48.47% and 55.43% had at least one decayed, missing and filled tooth, respectively. The mean number of decayed, missing, filled or DMFT for the whole sample were 2.4±3.6, 1.98±3.99, 1.79±2.45, 6.09±5.7, respectively. Decayed teeth were inversely correlated with socio-economic status (SES), education level, brushing frequency and milk consumption and positively correlated with grains, junk food and soda drinks consumption. Missing teeth were inversely correlated with SES, education level and brushing frequency, while positively correlated with age, BMI and caffeinated drink consumption. Conversely, filled teeth were positively correlated with age, BMI, SES and education level, while negatively correlated with grains and sugars in drinks.

**Conclusion** **:** The present study clarifies that age, BMI, SES, education level and brushing frequency are risk factors significantly associated with dental caries prevalence amongst Egyptian adults. Egyptian adults' dietary habits might lead to obesity, which indirectly causes dental caries rather than directly as in children.

## Introduction

Dental caries is the progressive destruction of the tooth structure by bacterial acids ^1^. It is considered the most prevalent chronic oral disease and the primary reason for tooth loss in adults. Dental caries has been estimated to affect almost every individual during their adult life, affecting an average of 5 to 10 teeth per each individual ^1– 3^. The prevalence of caries in a population is affected by numerous risk factors, such as sex, oral hygiene and dietary habits. Moreover, the prevalence of caries tends to increase with age as it is a cumulative process ^3– 6^. Tooth decay is particularly prevalent in developing countries owing to the dietary habits, socioeconomic conditions and a lack of education ^7^.

Data on the incidence of dental caries among Egyptian adults are scarce and are mostly grey literature, which makes this data hard to find. The last published report for the prevalence of caries among Egyptian adults was carried out by the World Health Organization (WHO) in collaboration with the Egyptian Ministry of Health in 2014 ^8^.

Therefore, the current study was carried out to investigate the prevalence of caries among Egyptian adults in correlation with different risk factors.

## Methods

### Subjects and methods

This study was carried out according to the regulations of the Research Ethics Committee of Faculty of Dentistry, Cairo University, Egypt (Approval:171217). Written informed consent was obtained from patients before participating in the study.

The subjects in this study were recruited between November 2017 to January 2018, from the outpatients' clinics of Faculty of Dentistry, Cairo University, which serves patients arriving from different parts of Egypt; in addition to two private dental clinics.

The inclusion criteria for the patients were: age, 18–74 years; either gender; ethnicity, Egyptian. The exclusion criteria were: history of radiotherapy and/or chemotherapy; subjects undergoing orthodontic therapy and patients who might not comply with study procedures (as judged by those who refused to answer all questions in the questionnaire ^9^).

### Sample size calculation

The sample size for caries in adults was estimated to be 264 individuals according to the following equation:

$$n^{\prime }=\frac{NZ^{\text{2}}2P(1-P)}{d^{2}(N-1)+Z^{2}P(1-P)}10.$$

n' = sample size with finite population correction, N = Egyptian adults population size (estimated by 45,000,000), Z = Z statistic for a level of confidence which is conventional (1.96). P = Expected prevalence (78.00%) and d = Precision (5% = (0.05)). The prevalence was estimated as 78%, as in India the caries prevalence was estimated to be 82.6–91.6% ^11^ and in Kosovo was 72.8% ^12^.

### Data collection and grouping

A total of 359 Egyptian adult patients were examined in this study. The collected data included name, age, gender, address, level of education; low (primary school or illiterate); moderate (diploma or high school); high (university), occupation and brushing frequency. A full assessment of the dietary habits was performed using a questionnaire ^9^.

Subjects were instructed to remove only their shoes while taking their anthropometric measurements. Weight was measured using a Beurer scale (Ulm, Germany) and their stature was measured to the nearest 0.1 cm using a stadiometer. Body mass index (BMI) was calculated using the formula: BMI = weight in kg / (height in m) ^2^. Patients were categorized according to their BMI as follows: underweight (<18.5 kg/m ^2^), normal (18.5–24.9 kg/m ^2^), overweight (25.0–29.9 kg/m ^2^) and obese (≥30.0 kg/m ^2^) ^13^.

Adults were classified according to their age as follows: BI (18–34 years old), BII (35–44 years old), BIII (45–64 years old) and BIV (65–75 years old) and were classified according to their socioeconomic status into low, moderate and high depending on their level of education, occupation and address ^14^.

### Oral examination

At the beginning, the authors (M.M.S., S.E., D.R., N.A., I.A.R.) were calibrated to avoid differences in observations and to reach a consensus. Oral examination was carried out according to WHO recommendations ^15^, as described in our previous study ^16^. All present teeth were examined for the presence of carious lesions. Teeth were carefully inspected for the presence of any lesion with a softened floor, undermined enamel, or softened wall, in a pit or fissure or on a smooth tooth surface. Tooth surface containing temporary filling or a permanent restoration but showing an area of decay (either primary or secondary caries) were also considered carious. DMFT index was used to measure the activity of caries, where D denotes decayed teeth, M is for missing teeth and F is for filled teeth ^17^.

### Statistical analysis

The statistical package used in this study is the R statistical package, version 3.3.1 (2016-06-21). For descriptive analyses, variables were described in terms of means ± standard deviations (SD), medians and ranges. For normality, Shapiro-Wilk test was applied to assess the normality of data. All data were not normally distributed. For comparative analysis, the non-parametric Kruskal-Wallis test was performed. Spearman’s correlation coefficient was calculated for correlation analysis. The significance level was verified at P ≤ 0.05. 

## Results

Raw data from the present study are available on figshare ^18^.

### Population profile

The mean number of decayed, missing, filled and DMFT for the whole sample were 2.4±3.6, 1.98±3.99, 1.79±2.45, 6.09±5.7, respectively. A total of 60.45%, 48.47% and 55.43% of participants had at least one decayed, missing or filled tooth, respectively. The prevalence of DMFT among participants was 86.63%. 

The number and percentage of adults in different categories in each studied parameter as well as comparisons between them are presented in Table 1. The number and percentage of participants that had decayed, missing, filled or DMFT in different categories in each studied parameter are presented in Table 2.

**Table 1.** Descriptive analysis of categorical variables and comparisons between proportions (N=359).

Parameter and categories		Number (%)	Pearson’s Chi-squared test	
			^2^	p-value
**Age**	**BI (18-34 years)**	185(51.53)	297.31	<0.0001 *
	**BII (35-44 years)**	81(22.56)		
	**BIII (45-64 years**	81(22.56)		
	**BIV (65-75 years)**	12(3.34)		
**Gender**	**Males**	150(41.78)	9.70	0.0018 *
	**Females**	209(58.22)		
**Body mass index**	**Underweight**	2(0.56)	134.8	<0.0001 *
	**Normal**	128(35.85)		
	**Overweight**	143(40.05)		
	**Obese**	84(23.53)		
**SES**	**Low**	95(26.46)	7.78	0.02 *
	**Moderate**	128(35.93)		
	**High**	134(37.6)		
**Level of education**	**Low**	46(12.81)	115.86	<0.0001 *
	**Moderate**	103(28.69)		
	**High**	210(58.5)		
**Brushing frequency**	**No brushing**	83(23.11)	114.57	<0.0001 *
	**Infrequent**	48(13.37)		
	**once daily**	106(29.52)		
	**Twice daily**	89(24.79)		
	**Three times**	32(8.91)		
	**Other**	1(0.27)		
**Reason for no brushing**	**Bleeding**	31 (31)	28.9	<0.0001 *
	**I don't know how to brush**	6 (6)		
	**I forget**	13 (13)		
	**I don't have time**	34 (34)		
	**Other**	16(16)		
**Dietary Habits**				
** -Bread**	**≤ 2 times/week**	13(3.62)	559.84	<0.0001 *
	**3-6 times/week**	15(4.18)		
	**1-6 times/day**	331(92.20)		
** -Other carbohydrates**	**≤ 2 times/week**	65(18.10)	214.77	<0.0001 *
	**>3-6 times/week**	44(12.26)		
	**>1-6 times/day**	250(69.64)		
** -Eggs**	**≤ 2 times/week**	236(65.74)	174.19	<0.0001 *
	**3-6 times/week**	78(21.73)		
	**1-6 times/day**	45(12.53)		
** -Fruits/Vegetables**	**≤ 2 times/week**	69(19.22)	161.6	<0.0001 *
	**3-6 times/week**	57(15.88)		
	**1-6 times/day**	233(64.90)		
** -Milk**	**≤ 2 times/week**	181(50.42)	107.32	<0.0001 *
	**3-6 times/week**	29(8.08)		
	**1-6 times/day**	149(41.50)		
** -Milk products**	**≤ 2 times/week**	107(29.81)	125.62	<.0.0001 *
	**3-6 times/week**	40(11.14)		
	**1-6 times/day**	212(59.05)		
** -Grains**	**≤ 2 times/week**	153(42.62)	59.12	<0.0001 *
	**3-6 times/week**	51(14.20)		
	**1-6 times/day**	155(43.18)		
** -Jam, Molasses and Honey**	**≤ 2 times/week**	264(73.54)	266.25	<0.0001 *
	**3-6 times/week**	30(8.36)		
	**1-6 times/day**	65(18.10)		
** -Candies**	**≤ 2 times/week**	249(69.36)	218.51	<0.0001 *
	**3-6 times/week**	32(8.91)		
	**1-6 times/day**	78(21.73)		
** -Crackers**	**≤ 2 times/week**	196(54.60)	113.99	<0.0001 *
	**3-6 times/week**	32(8.91)		
	**1-6 times/day**	131(36.49)		
** -Junk food**	**≤ 2 times/week**	240(66.85)	201.4	<0.0001 *
	**3-6 times/week**	25(6.96)		
	**1-6 times/day**	94(26.18)		
** -Chocolate**	**≤ 2 times/week**	261(72.70)	255.8	<0.0001 *
	**3-6 times/week**	31(8.64)		
	**1-6 times/day**	67(18.66)		
** -Soda**	**≤ 2 times/week**	211(58.77)	129.98	<0.0001 *
	**3-6 times/week**	35(9.75)		
	**1-6 times/day**	113(31.48)		
** -Juices**	**≤ 2 times/week**	223(62.11)	157.98	<0.0001 *
	**3-6 times/week**	30(8.36)		
	**1-6 times/day**	106(29.53)		
** -Citric juices**	**≤ 2 times/week**	274(76.32)	303.68	<0.0001 *
	**3-6 times/week**	25(6.96)		
	**1-6 times/day**	60(16.71)		
** -Caffeinated drinks**	**≤ 2 times/week**	47(13.09)	403.19	<0.0001 *
	**3-6 times/week**	14(3.90)		
	**1-6 times/day**	298(83)		

*Statistical significance at p-value ≤ *0.05.*

**Table 2.** Descriptive analysis of number and percentage of individuals that had untreated decayed, missing and filled teeth in different categories.

Parameter and categories		Number (%)			
		Decayed	Missing	Filled	DMFT
**Age**	**BI (18-34 years)**	108 (58.38)	52 (28.11)	94 (50.81)	184 (80)
	**BII (35-44 years)**	51 (62.96)	74 (58.82)	45 (55.56)	73 (90.12)
	**BIII (45-64 years**	53 (65.43)	63 (77.78)	51 (62.96)	78 (96.30)
	**BIV (65-75 years)**	5 (41.67)	11 (91.67)	9 (75)	12 (100)
**Gender**	**Males**	101 (67.33)	76 (50.67)	84 (56)	134 (89.33)
	**Females**	116 (55.50)	98 (46.89)	116 (55.50)	174 (83.25)
**Body mass index**	**Underweight**	1 (50)	0 (0)	1 (50)	2 (100)
	**Normal**	78 (60.94)	46 (35.94)	61 (47.66)	108 (84.38)
	**Overweight**	87 (60.48)	69 (48.25)	83 (58.04)	118 (82.52)
	**Obese**	48 (57.14)	59 (70.24)	54 (64.29)	79 (94.05)
**SES**	**Low**	77 (81.05)	67 (70.53)	42 (44.21)	89 (93.68)
	**Moderate**	72 (55.81)	54 (41.86)	72 (55.81)	108 (83.72)
	**High**	68 (50.75)	53 (39.55)	86 (64.18)	113 (84.33)
**Level of education**	**Low**	74 (71.84)	32 (31.07)	21 (20.39)	42 (40.78)
	**Moderate**	52 (50.49)	48 (46.6)	40 (38.83)	75 (72.82)
	**High**	113 (53.81)	79 (37.62)	133 (63.33)	176 (83.81)
**Brushing frequency**	**No brushing**	60 (72.29)	51 (61.45)	43 (51.81)	81 (97.59)
	**Infrequent**	36 (75)	29 (60.42)	28 (58.33)	42 (87.50)
	**once daily**	61 (57.55)	59 (55.66)	60 (56.60)	92 (86.79)
	**Twice daily**	48 (53.93)	25 (28.09)	53 (59.55)	73 (82.02)
	**Three times**	10 (31.25)	8 (25)	14 (43.75)	20 (62.5)
	**Other**	0 (0)	1 (100)	1 (100)	1(100)
**Dietary habits**					
** -Bread**	**≤ 2 times/week**	9 (69.23)	5 (38.46)	8 (61.54)	11 (84.62)
	**3-6 times/week**	7 (64.67)	3 (20)	8 (53.33)	11 (73.33)
	**1-6 times/day**	201 (60.73)	165 (49.85)	187 (56.5)	289 (87.31)
** -Other carbohydrates**	**≤ 2 times/week**	37 (56.92)	35 (53.85)	35 (53.85)	56 (86.15)
	**3-6 times/week**	24 (54.55)	19 (43.18)	25 (56.82)	39 (88.64)
	**1-6 times/day**	155 (62)	120 (48)	140 (56)	215 (86)
** -Eggs**	**≤ 2 times/week**	156 (66.1)	116 (49.15)	130 (55.08)	202 (85.59)
	**3-6 times/week**	39 (50)	37 (47.44)	43 (55.13)	66 (84.62)
	**1-6 times/day**	31 (68.89)	20 (44.44)	26 (57.78)	41 (91.11)
** -Fruits/Vegetables**	**≤ 2 times/week**	43 (62.32)	35 (50.72)	39 (56.52)	62 (89.86)
	**3-6 times/week**	31 (54.39)	30 (52.63)	38 (66.67)	51 (89.47)
	**1-6 times/day**	140 (60.34)	107 (46.12)	121 (52.16)	195 (84.05)
** -Milk**	**≤ 2 times/week**	120 (66.3)	88 (48.62)	100 (55.25)	159 (87.85)
	**3-6 times/week**	20 (68.97)	16 (55.17)	21 (72.41)	27 (93.1)
	**1-6 times/day**	78 (51.32)	70 (46.05)	81 (53.29)	127 (83.55)
** -Milk products**	**≤ 2 times/week**	66 (61.88)	56 (52.34)	52 (48.6)	92 (85.98)
	**3-6 times/week**	24 (60)	20 (50)	29 (72.5)	37 (92.5)
	**1-6 times/day**	126 (59.43)	98 (46.23)	119 (56.13)	182 (85.85)
** -Grains**	**≤ 2 times/week**	82 (53.59)	76 (49.67)	86 (56.21)	125 (81.7)
	**3-6 times/week**	30 (58.82)	28 (54.9)	39 (76.47)	48 (94.12)
	**1-6 times/day**	105 (67.74)	70 (45.16)	75 (48.39)	137 (88.39)
** -Sugar in drinks**	**≤ 2 times/week**	42 (64.62)	29 (44.62)	36 (55.38)	59 (90.77)
	**3-6 times/week**	6 (50)	5 (41.76)	10 (83.33)	12 (100)
	**1-6 times/day**	168 (59.57)	139 (49.29)	150 (53.19)	200 (70.92)
** -Sugar not in drinks**	**≤ 2 times/week**	149 (60.32)	131 (53.04)	137 (55.47)	216 (87.45)
	**3-6 times/week**	9 (56.25)	6 (37.5)	13 (81.25)	16 (100)
	**1-6 times/day**	59 (61.46)	36 (37.5)	50 (52.08)	79 (82.29)
** -Jam, Molasses and Honey**	**≤ 2 times/week**	164 (62.36)	134 (50.95)	135 (51.33)	227 (86.31)
	**3-6 times/week**	17 (56.67)	16 (53.33)	23 (76.67)	28 (93.33)
	**1-6 times/day**	37 (56.92)	24 (36.92)	42 (64.62)	56 (86.15)
** -Candies**	**≤ 2 times/week**	152 (61.04)	140 (56.22)	144 (57.83)	220 (88.35)
	**3-6 times/week**	20 (62.5)	9 (28.13)	18 (56.25)	27 (84.38)
	**1-6 times/day**	59 (61.46)	36 (37.5)	50 (52.08)	79 (82.29)
** -Crackers**	**≤ 2 times/week**	118 (59.6)	109 (55.05)	117 (59.09)	178 (89.9)
	**3-6 times/week**	17 (53.13)	16 (50)	20 (62.5)	27 (84.38)
	**1-6 times/day**	83 (63.85	49 (37.69)	64 (49.23)	107 (82.31)
** -Junk food**	**≤ 2 times/week**	138 (57.5)	122 (50.83)	137 (57.08)	217 (90.42)
	**3-6 times/week**	13 (52)	15 (60)	19 (76)	23 (92)
	**1-6 times/day**	65 (69.15)	37 (39.36)	44 (46.81)	81 (86.17)
** -Chocolate**	**≤ 2 times/week**	158 (60.54)	143 (54.79)	134 (54.79)	239 (91.57)
	**3-6 times/week**	18 (58.06)	12 (38.71)	18 (58.06)	26 (83.87)
	**1-6 times/day**	36 (53.73)	19 (28.36)	39 (58.21)	65 (97.01)
** -Soda**	**≤ 2 times/week**	119 (56.4)	112 (53.08)	112 (53.08)	184 (87.2)
	**3-6 times/week**	26 (74.29)	20 (57.14)	27 (77.14)	32 (91.43)
	**1-6 times/day**	71 (62.83)	42 (37.17)	61 (53.98)	84.07)
** -Juices**	**≤ 2 times/week**	135 (60.54)	125 (56.05)	126 (56.5)	199 (89.24)
	**3-6 times/week**	14 (46.67)	12 (40)	19 (63.33)	24 (80)
	**1-6 times/day**	67 (63.21)	37 (34.91)	53 (50)	87 (82.08)
** -Citric juices**	**≤ 2 times/week**	163 (59.49)	138 (50.36)	150 (54.74)	237 (86.5)
	**3-6 times/week**	15 (60)	11 (44)	16 (64)	22 (88)
	**1-6 times/day**	39 (65)	25 (41.67)	34 (56.67)	52 (86.67)
** -Caffeinated drinks**	**≤ 2 times/week**	25 (53.19)	16 (34.04)	22 (46.81)	37 (78.72)
	**3-6 times/week**	10 (71.43)	8 (57.14)	9 (64.29)	14 (100)
	**1-6 times/day**	181 (60.74)	150 (50.34)	169 (56.71)	260 (87.25)

### Decayed teeth in correlation to different caries risk factors

**Figure 1.**
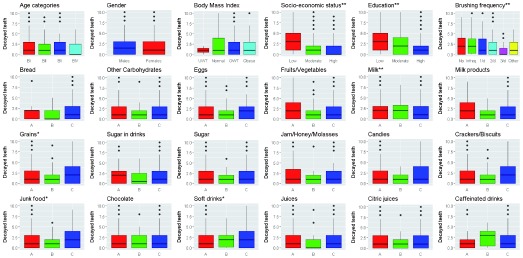
Decayed permanent teeth and different risk factors. N= 359: *positive correlations; **inverse correlations. A: ≤2 times/week, B: 3–6 times/week, C: 1–6 times/day.

As shown in Table 3 and Figure 1, despite the highest means of decayed teeth were recorded in adults aged 65–75 years, in males and in adults of normal weight (3.42±7.39, 2.61±3.68 and 2.83±3.77, respectively), the differences in medians were statistically insignificant (p≥0.05) and there was no correlation between age, gender or BMI and decayed teeth (Spearman’s rho=-0.04, 0.08 and -0.07, respectively; p≥0.05).

**Table 3.** Decayed teeth and different risk factors (N= 359).

Parameter and categories		Mean (SD)	Median (Range)	Correlation		K-W test
				rho	p-value *	p-value
**Age**	**BI (18-34 years)**	2.54(3.27)	2(0–16)	-0.04	0.498	0.8434
	**BII (35-44 years)**	2.12(3.13)	1(0–17)			
	**BIII (45-64 years**	2.25(3.99)	1(0–32)			
	**BIV(65-75 years)**	3.42(7.39)	0(0–26)			
**Gender**	**Males**	2.61(3.68)	2(0–26)	-0.08	0.1201	0.12
	**Females**	2.26(3.55)	1(0–32)			
**Body mass index**	**Underweight**	1(1.41)	1(0–2)	-0.07	0.177	0.502
	**Normal**	2.83(3.77)	1(0–17)			
	**Overweight**	2.42(4.11)	2(0–32)			
	**Obese**	1.7(2.03)	1(0–10)			
**SES**	**Low**	4.08(4.63)	3(0–32)	-0.28	<0.0001 *	<0.0001 *
	**Moderate**	1.87(2.79)	1(0–15)			
	**High**	1.73(3.10)	0.5(0–26)			
**Level of education**	**Low**	4.78(5.97)	3(0–32)	-0.24	<0.0001 *	<0.0001 *
	**Moderate**	2.34(2.51)	2(0–10)			
	**High**	1.91(3.16)	1(0–26)			
**Brushing frequency**	**No brushing**	4.26(5.76)	3(0–32)	-0.26	<0.0001 *	0.0001 *
	**Infrequent**	2.47(2.47)	2.5(0–10)			
	**once daily**	2.17(2.90)	1(0–15)			
	**Twice daily**	1.76(2.44)	1(0–10)			
	**Three times**	0.96(1.40)	0(0–5)			
**Dietary Habits**						
** -Bread**	**≤ 2 times/week**	1.85(2.38)	2(0–9)	0.06	0.2491	0.348
	**3-6 times/week**	1.13(1.51)	0(0–5)			
	**1-6 times/day**	2.48(3.70)	1(0–32)			
** -Other carbohydrates**	**≤ 2 times/week**	1.98(2.68)	1(0–12)	0.07	0.1575	0.3128
	**3-6 times/week**	2.59(5.58)	1(0–32)			
	**1-6 times/day**	2.48(3.38)	2(0–26)			
** -Eggs**	**≤ 2 times/week**	2.57(3.76)	1(0–32)	-0.03	0.5291	0.0266 *
	**3-6 times/week**	1.49(2.04)	1(0–8)			
	**1-6 times/day**	3.11(2.54)	2(0–26)			
** -Fruits/Vegetables**	**≤ 2 times/week**	2.42(2.74)	2(0–12)	-0.01	0.8853	0.3137
	**3-6 times/week**	1.84(2.80)	1(0–16)			
	**1-6 times/day**	2.54(3.98)	1(0–32)			
** -Milk**	**≤ 2 times/week**	2.94(4.33)	2(0–32)	-0.15	0.0037 *	0.0095 *
	**3-6 times/week**	2.45(2.76)	2(0–12)			
	**1-6 times/day**	1.79(2.59)	1(0–13)			
** -Milk products**	**≤ 2 times/week**	2.80(4.50)	2(0–32)	-0.03	0.5113	0.5401
	**3-6 times/week**	2.15(3.53)	1(0–16)			
	**1-6 times/day**	2.25(3.07)	1(0–17)			
** -Grains**	**≤ 2 times/week**	2.19(3.28)	1(0–17)	0.13	0.0143 *	0.0131 *
	**3-6 times/week**	1.55(2.31)	1(0–12)			
	**1-6 times/day**	2.90(4.16)	2(0–32)			
** -Sugar in drinks**	**≤ 2 times/week**	2.74(4.12)	2(0–26)	-0.03	0.5519	0.5306
	**3-6 times/week**	1.67(2.35)	0.5(0–6)			
	**1-6 times/day**	2.36(3.52)	1(0–32)			
** -Sugar not in drinks**	**≤ 2 times/week**	2.48(3.93)	1(0–32)	0.01	0.8317	0.6475
	**3-6 times/week**	1.44(1.75)	1(0–6)			
	**1-6 times/day**	2.38(2.87)	1.5(0–12)			
** -Jam, Molasses and Honey**	**≤ 2 times/week**	2.49(3.69)	1.5(0–32)	-0.06	0.2471	0.3784
	**3-6 times/week**	1.70(2.68)	1(0–12)			
	**1-6 times/day**	2.37(3.62)	1(0–16)			
** -Candies**	**≤ 2 times/week**	2.51(3.87)	1(0–32)	-0.02	0.6968	0.6133
	**3-6 times/week**	1.38(1.36)	1(0–4)			
	**1-6 times/day**	2.50(2.32)	1(0–16)			
** -Crackers**	**≤ 2 times/week**	2.35(4.02)	1(0–32)	0.08	0.1286	0.1388
	**3-6 times/week**	1.63(2.11)	1(0–8)			
	**1-6 times/day**	2.67(3.20)	2(0–16)			
** -Junk food**	**≤ 2 times/week**	2.14(3.57)	1(0–32)	0.15	0.0058 *	0.0035 *
	**3-6 times/week**	1.44(1.87)	1(0–6)			
	**1-6 times/day**	3.34(3.87)	2(0–17)			
** -Chocolate**	**≤ 2 times/week**	2.41(3.79)	1(0–32)	0.01	0.7924	0.9448
	**3-6 times/week**	2.29(3.30)	1(0–16)			
	**1-6 times/day**	2.43(3)	1(0–10)			
** -Soda drinks**	**≤ 2 times/week**	2.01(3.16)	1(0–26)	0.13	0.0121 *	0.0434 *
	**3-6 times/week**	2.11(2.39)	2(0–12)			
	**1-6 times/day**	3.22(4.47)	2(0–32)			
** -Juices**	**≤ 2 times/week**	2.40(3.39)	1(0–26)	-0.01	0.8482	0.2614
	**3-6 times/week**	2.57(6.23)	0(0–32)			
	**1-6 times/day**	2.36(3.03)	1.5(0–15)			
** -Citric juices**	**≤ 2 times/week**	2.51(3.87)	1(0–32)	-0.002	0.9672	0.4813
	**3-6 times/week**	1.40(1.80)	1(0–8)			
	**1-6 times/day**	2.35(2.78)	1(0–10)			
** -Caffeinated drinks**	**≤ 2 times/week**	1.83(2.98)	1(0–15)	0.04	0.4389	0.1366
	**3-6 times/week**	2.79(2.19)	3(0–6)			
	**1-6 times/day**	2.48(3.74)	1(0–32)			

The correlation coefficient, rho, ranges from -1 to +1. Where 1= perfect positive correlation, 0=no correlation, -1 = perfect negative (inverse) correlation. *Statistical significance at p-value ≤ *0.05*.

Adults with low SES, low education level and who do not brush their teeth had the highest mean number of decayed teeth (4.08±4.63, 4.78±5.97 and 4.26±5.76, respectively). The differences in medians were statistically significant (p≤0.0001). SES, education level and brushing frequency were inversely correlated with number of decayed teeth (Spearman’s rho= -0.28, -0.24, and -0.26, respectively; p≤0.0001).

Adults who consume bread, eggs, fruits/vegetables, grains, crackers, junk food, chocolate, soft drinks and caffeinated drinks 1–6 times per day had the highest mean number of decayed teeth (2.48±3.70, 3.11±2.54, 2.54±3.98, 2.90±4.16, 2.67±3.2, 3.34±3.87, 2.43±3, 3.22±4.47 and 2.48±3.74, respectively). Milk consumption was significantly inversely correlated with decayed teeth (rho= -0.15, p=0.0037), whereas consumption of grains, junk food and soft drinks were significantly positively correlated (rho=0.13, 0.15 and 0.13; p=0.0143, 0.0058 and 0.0121, respectively). The differences in medians for decayed teeth in all dietary elements were statistically insignificant except for eggs, milk, grains and junk food.

### Missing teeth and different caries risk factors

**Figure 2.**
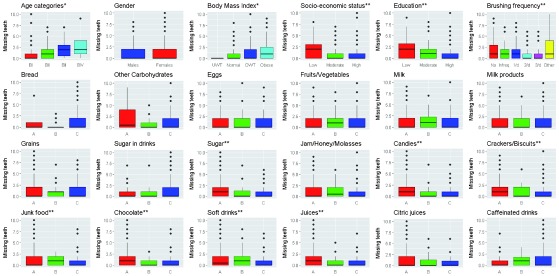
Missing permanent teeth and different risk factors. N= 359: *positive correlations; **inverse correlations. A: ≤2 times/week, B: 3–6 times/week, C: 1–6 times/day.

As revealed in Table 4 and Figure 2, the highest mean number of missing teeth were detected in adults aged 65–75 years, besides in obese adults (6.08±6.64 and 3.51±5.56, respectively). The differences in medians were statistically significant (p≤0.0001). Age and BMI were directly correlated with number of missing teeth (Spearman’s rho=0.44 and 0.25, respectively; p≤0.0001).

**Table 4.** Missed teeth and different risk factors (N=359).

Parameter and categories		Mean (SD)	Median (Range)	Correlation		K-W test
				rho	p-value *	p-value
**Age**	**BI (18-34 years)**	0.85 (1.98)	0 (0–11)	0.44	<0.0001 *	<0.0001 *
	**BII (35-44 years)**	1.91 (3.54)	1 (0–27)			
	**BIII (45-64 years**	4.05 (5.82)	2(0–26)			
	**BIV (65-75 years)**	6.08 ( 6.64)	3.5 (0–22)			
**Gender**	**Males**	2.07 (4.38)	1 (0–27)	-0.02	0.7748	0.7744
	**Females**	1.92 (3.69)	0 (0–26)			
**Body mass index**	**Underweight**	0 (0)	0(0)	0.25	<0.0001 *	<0.0001 *
	**Normal**	1.5 (3.60)	0 (0–22)			
	**Overweight**	1.57 (2.94)	0 (0–21)			
	**Obese**	3.51 (5.56)	2 (0–27)			
**SES**	**Low**	3.23 (4.84)	2 (0–27)	-0.25	<0.0001 *	<0.0001 *
	**Moderate**	1.62 (3.92)	0 (0–26)			
	**High**	1.48 (3.18)	0 (0–22)			
**Level of education**	**Low**	3.78 (5.09)	2 (0–21)	-0.30	<0.0001 *	<0.0001 *
	**Moderate**	2.72 (4.94)	1 (0–27)			
	**High**	1.23 (2.87)	0 (0–22)			
**Brushing frequency**	**No brushing**	3.34 (5.57)	1 (0–27)	-0.23	<0.0001 *	0.0003 *
	**Infrequent**	1.61 (2.02)	1 (0–7)			
	**once daily**	2.01 (3.70)	1 (0–22)			
	**Twice daily**	1.46 (3.97)	0 (0–26)			
	**Three times**	0.96 ( 1.71)	0 (0–6)			
**Dietary Habits**						
** -Bread**	**≤ 2 times/week**	2.31 (4.35)	0 (0–14)	0.10	0.0523	0.0687
	**3-6 times/week**	0.40 (0.91)	0 (0–3)			
	**1-6 times/day**	2.04 (4.05)	1 (0–27)			
** -Other carbohydrates**	**≤ 2 times/week**	3 (4.44)	1 (0–22)	-0.05	0.3909	0.0319
	**3-6 times/week**	0.68 (1.03)	0 (0–5)			
	**1-6 times/day**	1.95 (4.13)	0 (0–27)			
** -Eggs**	**≤ 2 times/week**	1.94 (3.90)	0 (0–26)	-0.01	0.9242	0.8232
	**3-6 times/week**	1.45 (2.44)	0 (0–11)			
	**1-6 times/day**	3.11 (6.01)	0 (0–27)			
** -Fruits/Vegetables**	**≤ 2 times/week**	2.09 (3.82)	1 (0–21)	-0.03	0.6172	0.8806
	**3-6 times/week**	1.58 (3.11)	1 (0–21)			
	**1-6 times/day**	2.05 (4.23)	0 (0–27)			
** -Milk**	**≤ 2 times/week**	1.79 (3.43)	0 (0–21)	0.01	0.9229	0.8256
	**3-6 times/week**	1.72 (2.52)	1 (0–11)			
	**1-6 times/day**	2.27 (4.78)	0 (0–27)			
** -Milk products**	**≤ 2 times/week**	2.34 ( 4.44)	1 (0–27)	-0.07	0.2099	0.4373
	**3-6 times/week**	1.60 (2.69)	0.5 (0–11)			
	**1-6 times/day**	1.88 (3.96)	0 (0–26)			
** -Grains**	**≤ 2 times/week**	2.13 (4.13)	0 (0–26)	-0.05	0.3611	0.6173
	**3-6 times/week**	2.06 (4.06)	1 (0–22)			
	**1-6 times/day**	1.81 (3.85)	0 (0–27)			
** -Sugar in drinks**	**≤ 2 times/week**	1.15 (2.07)	0 (0–11)	0.09	0.1068	0.2488
	**3-6 times/week**	1 (2)	0 (0–7)			
	**1-6 times/day**	2.22 (4.35)	0 (0–27)			
** -Sugar not in drinks**	**≤ 2 times/week**	2.33 (4.40)	1 (0–27)	-0.16	0.0026 *	0.0105 *
	**3-6 times/week**	1.06 (1.95)	0 (0–7)			
	**1-6 times/day**	1.24 (2.87)	0 (0–22)			
** -Jam, Molasses and Honey**	**≤ 2 times/week**	2.04 (4.09)	1 (0–27)	-0.07	0.2067	0.2771
	**3-6 times/week**	1.97 (3.23)	1 (0–13)			
	**1-6 times/day**	1.77 (3.94)	0 (0–22)			
** -Candies**	**≤ 2 times/week**	2.47 (4.42)	1 (0–27)	-0.25	<0.0001 *	<0.0001 *
	**3-6 times/week**	0.63 (1.41)	0 (0–7)			
	**1-6 times/day**	1 (2.80)	0 (0–22)			
** -Crackers**	**≤ 2 times/week**	2.57 (4.62)	1 (0–26)	-0.18	0.0007 *	0.0034 *
	**3-6 times/week**	1.47 (2.69)	0.5 (0–14)			
	**1-6 times/day**	1.24 (2.99)	0 (0–27)			
** -Junk food**	**≤ 2 times/week**	2.49 (4.68)	1 (0–27)	-0.13	0.0122 *	0.03611 *
	**3-6 times/week**	0.96 (0.98)	1 (0–3)			
	**1-6 times/day**	0.97 (1.65)	0 (0–8)			
** -Chocolate**	**≤ 2 times/week**	2.32 (4.21)	1 (0–27)	-0.22	<0.0001 *	0.0002 *
	**3-6 times/week**	0.68 (1.01)	0 (0–3)			
	**1-6 times/day**	1.27 (3.80)	0 (0–26)			
** -Soda drinks**	**≤ 2 times/week**	2.40 (4.45)	1 (0–26)	-0.16	0.0029 *	0.006 *
	**3-6 times/week**	1.97 (3.27)	1 (0–14)			
	**1-6 times/day**	1.21 (3.11)	0 (0–27)			
** -Juices**	**≤ 2 times/week**	2.42 (4.18)	1 (0–26)	-0.21	<0.0001 *	0.0003 *
	**3-6 times/week**	1.10 (2.22)	0 (0–11)			
	**1-6 times/day**	1.32 (3.87)	0 (0–27)			
** -Citric juices**	**≤ 2 times/week**	2.06 (3.92)	1 (0–26)	-0.08	0.1302	0.3176
	**3-6 times/week**	1.80 (4.47)	0 (0–22)			
	**1-6 times/day**	1.70 (4.18)	0 (0–27)			
** -Caffeinated drinks**	**≤ 2 times/week**	1.49 (4.35)	0 (0–26)	0.11	0.0441 *	0.0814
	**3-6 times/week**	1.21 (1.48)	1 (0–4)			
	**1-6 times/day**	2.10 (4.01)	1 (0–27)			

The correlation coefficient, rho, ranges from -1 to +1. Where 1= perfect positive correlation, 0=no correlation, -1 = perfect negative (inverse) correlation. *Statistical significance at p-value ≤ *0.05*.

However, males had a higher mean missing teeth than females (2.07±4.38), the difference in medians was statistically insignificant (p≥0.05) and there was no correlation between gender and missing teeth (Spearman’s rho= -0.02, p≥0.05).

Adults with low SES, low education level and those who don’t brush their teeth had the highest means missing teeth (3.23±4.84, 3.78±5.09 and 3.34±5.57, respectively). The differences in medians were statistically significant (p≤0.0001). SES, education level and brushing frequency were inversely correlated with number of missing teeth (Spearman’s rho= -0.25, -0.3 and -0.23, respectively; p≤0.0001).

Adults who consume eggs, milk, sugar in drinks and caffeinated drinks 1–6 times per day had the highest means missing teeth (3.11±6.01, 2.27±4.78, 2.22±4.35 and 2.10±4.01, respectively); while those who consume bread, other carbohydrates, fruits/vegetables, milk products, grains, sugar, jam, candies, crackers, junk food, chocolate, soft drinks, juices and citric juices less than or equal to two times a week had the highest means of missing teeth (2.31±4.35, 3±4.44, 2.09±3.82, 2.34±4.44, 2.13±4.13, 2.33±4.4, 2.04±4.09, 2.47±4.42, 2.57±4.62, 2.49±4.68, 2.32±4.21, 2.40±4.45, 2.42±4.18 and 2.06±3.92, respectively). The differences in medians for missing teeth in all parameters were statistically insignificant except for other carbohydrates, sugar not in drinks, candies, crackers, junk food, chocolate, soft drinks and juices. Consumption of sugars not included in drinks, candies, crackers, junk food, chocolate, soft drinks, juices and caffeinated drinks were inversely correlated with missing teeth.

### Filled teeth and different caries risk factors

**Figure 3.**
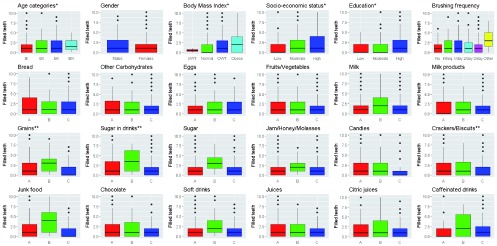
Filled permanent teeth and different risk factors. N= 359: *positive correlations; **inverse correlations. A: ≤2 times/week, B: 3–6 times/week, C: 1–6 times/day.

As seen in Table 5 and Figure 3, adults aged 65–75 years and obese adults had the highest mean numbers of filled teeth (1.83±1.64 and, 3.51±5.56, respectively). The difference in medians was statistically insignificant for age (p->0.05), while was statistically significant for BMI (p=0.0223). Age and BMI were directly correlated with number of filled teeth (Spearman’s rho=0.13 and 0.16, and p=0.0138 and, 0.002, respectively).

**Table 5.** Filled teeth and different risk factors (N=359).

Parameter and categories		Mean (SD)	Median (Range)	Correlation		K-W test
				rho	p-value *	p-value
**1- Age**	**BI (18-34 years)**	1.51(2.15)	1(0–10)	0.13	0.0138 *	0.0888
	**BII (35-44 years)**	1.76(2.48)	1 (0–13)			
	**BIII (45-64 years**	2.44(3)	2(0–11)			
	**BIV (65-75 years)**	1.83(1.64)	1.5(0–5)			
**2- Gender**	**Males**	1.81(2.4)	1(0–13)	-0.02	0.7371	0.7366
	**Females**	1.78(2.49)	1(0–11)			
**3- Body Mass Index**	**Underweight**	0.5(0.71)	0.5(0–1)	0.16	0.002 *	0.0223 *
	**Normal**	1.38(2.1)	0(0–10)			
	**Overweight**	1.89(2.62)	1(0–13)			
	**Obese**	2.31(2.59)	2(0–11)			
**4- SES**	**Low**	1.04(1.64)	**0(0–10)**	0.18	0.0004 *	0.0016 *
	**Moderate**	1.86(2.51)	1(0–11)			
	**High**	2.26(2.74)	1 (0–13)			
**5- Level of education**	**Low**	0.96(1.3)	0(0–5)	0.21	<0.0001 *	0.0002 *
	**Moderate**	1.25(2.03)	0(0–10)			
	**High**	2.24(2.72)	1 (0–13)			
**6- Brushing frequency**	**No brushing**	1.39(1.79)	1(0–6)	0.04	0.4322	0.7093
	**Infrequent**	1.82(2.48)	1(0–9)			
	**once daily**	2.01(2.69)	1(0–11)			
	**Twice daily**	1.84(2.63)	1(0–13)			
	**Three times**	1.72(2.36)	0(0–9)			
	**Other**	3.5(3.42)	3(0–8)			
**7-Dietary Habits**						
** -Bread**	**≤ 2 times/week**	2.62(3.36)	1(0–9)	-0.02	0.6597	0.7676
	**3-6 times/week**	1.93(2.91)	1(0–10)			
	**1-6 times/day**	1.76(2.39)	1(0–13)			
** -Other carbohydrates**	**≤ 2 times/week**	2.09(2.83)	1(0–13)	-0.04	0.4946	0.7739
	**3-6 times/week**	2.07(2.87)	1(0–11)			
	**1-6 times/day**	1.66(2.26)	1(0–11)			
** -Eggs**	**≤ 2 times/week**	1.75(2.37)	1(0–13)	0.01	0.8744	0.9069
	**3-6 times/week**	2.01(2.8)	1(0–11)			
	**1-6 times/day**	1.64(2.22)	1(0–9)			
** -Fruits/Vegetables**	**≤ 2 times/week**	1.94(2.72)	1(0–10)	-0.07	0.2015	0.1702
	**3-6 times/week**	2.18(2.52)	1(0–11)			
	**1-6 times/day**	1.66(2.35)	1(0–13)			
** -Milk**	**≤ 2 times/week**	1.79(2.45)	1(0–11)	-0.02	0.6665	0.1161
	**3-6 times/week**	2.48(2.56)	2(0–10)			
	**1-6 times/day**	1.66(2.42)	1(0–13)			
** -Milk products**	**≤ 2 times/week**	1.7(2.42)	0(0–10)	0.02	0.7511	0.1836
	**3-6 times/week**	2.05(2.1)	1(0–7)			
	**1-6 times/day**	1.79(2.53)	1(0–13)			
** -Grains**	**≤ 2 times/week**	2.11(2.91)	1(0–13)	-0.14	0.0102 *	<0.0001 *
	**3-6 times/week**	3(2.69)	3(0–9)			
	**1-6 times/day**	1.08(1.47)	0(0–7)			
** -Sugar in drinks**	**≤ 2 times/week**	2.45(3.18)	1(0–11)	-0.11	0.0414 *	0.0125 *
	**3-6 times/week**	3.67(2.9)	3.5(0–8)			
	**1-6 times/day**	1.56(2.17)	1(0–13)			
** -Sugar not in drinks**	**≤ 2 times/week**	1.76(2.46)	1(0–13)	-0.004	0.9938	0.009
	**3-6 times/week**	3.38(2.47)	3(0–8)			
	**1-6 times/day**	1.61(2.36)	1(0–11)			
** -Jam, Molasses and Honey**	**≤ 2 times/week**	1.68(2.39)	1(0–11)	0.1	0.051	0.0453 *
	**3-6 times/week**	2.3(2.14)	2(0–7)			
	**1-6 times/day**	2.02(2.8)	1(0–13)			
** -Candies**	**≤ 2 times/week**	1.85(2.33)	1(0–11)	-0.01	0.0821	0.0861
	**3-6 times/week**	2.28(2.96)	1.5(0–11)			
	**1-6 times/day**	1.42(2.57)	0(0–13)			
** -Crackers**	**≤ 2 times/week**	2.02(2.66)	1(0–13)	-0.11	0.0435 *	0.0731
	**3-6 times/week**	2(2.31)	1(0–8)			
	**1-6 times/day**	1.4(2.32)	0(0–10)			
** -Junk food**	**≤ 2 times/week**	1.87(2.53)	1(0–13)	-0.08	0.134	0.0001 *
	**3-6 times/week**	3.72(3.1)	4(0–10)			
	**1-6 times/day**	1.1(1.63)	0(0–7)			
** -Chocolate**	**≤ 2 times/week**	1.86(2.57)	1(0–13)	-0.01	0.8298	0.7281
	**3-6 times/week**	2.13(2.67)	1(0–10)			
	**1-6 times/day**	1.39(1.74)	1(0–8)			
** -Soda drinks**	**≤ 2 times/week**	1.79(2.58)	1(0–13)	0.01	0.8692	0.0162 *
	**3-6 times/week**	2.77(2.78)	2(0–10)			
	**1-6 times/day**	1.5(1.98)	1(0–8)			
** -Juices**	**≤ 2 times/week**	2.03(2.71)	1(0–13)	-0.09	0.081	0.1201
	**3-6 times/week**	1.25(1.7)	1(0–8)			
	**1-6 times/day**	1.97(2.39)	1(0–7)			
** -Citric juices**	**≤ 2 times/week**	1.79(2.45)	1(0–13)	-0.003	0.9551	0.303
	**3-6 times/week**	2.64(3.07)	1(0–10)			
	**1-6 times/day**	1.45(2.1)	1(0–9)			
** -Caffeinated drinks**	**≤ 2 times/week**	1.74(2.84)	0(0–13)	0.01	0.8177	0.2232
	**3-6 times/week**	2.93(2.97)	2(0–8)			
	**1-6 times/day**	1.75(2.35)	1(0–11)			

*P<0.05.

Males had a higher mean number of filled teeth than females (1.81±2.4), while adults who don’t brush their teeth had the lowest mean number of filled teeth (1.39±1.79). The differences in medians were statistically insignificant (p>0.05) and there was no correlation between gender or brushing frequency and number of filled teeth (Spearman’s rho=-0.02 and 0.04, respectively; p>0.05).

Adults with high SES and high education levels had the highest mean number of filled teeth (2.26±2.74 and 2.24±2.72, respectively); the differences in medians were statistically significant (p=0.0016 and 0.0002, respectively). SES and education level were directly correlated with filled teeth (Spearman’s rho=0.18 and 0.21; p=0.0004 and <0.0001, respectively).

Adults who consume eggs, fruits/vegetables, milk, milk products, grains, sugar in drinks, sugar not in drinks, jam, candies, junk food, chocolate, soft drinks, citric juices and caffeinated drinks 3–6 times per week had the highest means of filled teeth (2.01±2.8, 2.18±2.52, 2.48±2.56, 2.05±2.1, 3±2.69, 3.67± 2.9, 3.38±2.47, 2.3±2.14, 2.28±2.96, 3.72±3.1, 2.13±2.67, 2.77±2.78, 2.64±3.07 and 2.93±2.97, respectively). Moreover, adults who consume bread, other carbohydrates, crackers and juices less than or equal to two times a week had the highest mean numbers of filled teeth (2.62±3.36, 2.09±2.83, 2.02±2.66 and, 2.03±2.71, respectively).

The differences in medians for filled teeth regarding all dietary elements were statistically insignificant except for grains, sugar in drinks, sugar not in drinks, jam, junk food and soft drinks. Grains, sugar in drinks and crackers were inversely correlated with filled teeth, while jams were borderline positively correlated.

### DMFT and different caries risk factors

**Figure 4.**
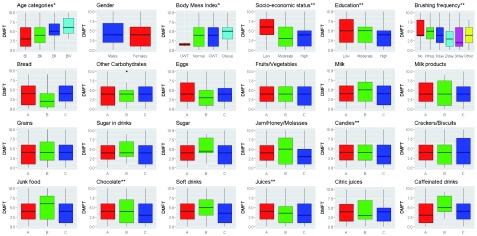
DMFT (caries) in permanent teeth and different risk factors. N= 359: *positive correlations; **inverse correlations. A: ≤2 times/week, B: 3–6 times/week, C: 1–6 times/day.

Table 6, Figure 4 show that the highest means DMFT was in adults aged 65–75 years and obese adults (11.42±7.63 and 7.4±5.89, respectively). The differences in medians were statistically significant (p≤0.0001). Age and BMI were directly correlated with DMFT (Spearman’s rho=0.33 and 0.15; p≤0.0001 and 0.0053, respectively).

**Table 6.** DMFT and different risk factors (N= 359).

Parameter and categories		Mean (SD)	Median (Range)	Correlation		K-W test
				Rho	p-value *	p-value
**Age**	**BI (18-34 years)**	4.71(4.78)	4(0–30)	0.33	<0.0001 *	<0.0001 *
	**BII (35-44 years)**	5.8(4.95)	5(0–27)			
	**BIII (45-64 years**	8.74(6.67)	7(0–32)			
	**BIV (65-75 years)**	11.42(7.63)	10(0–26)			
**Gender**	**Males**	6.5 (5.47)	5(0–27)	-0.09	0.0773	0.207
	**Females**	5.79(5.85)	4(0–32)			
**Body mass index**	**Underweight**	1.5(0.71)	1.5(1–2)	0.15	0.0053 *	0.0118 *
	**Normal**	5.62(5.64)	4(0–29)			
	**Overweight**	5.8(5.61)	4(0–32)			
	**Obese**	7.4(5.89)	6(0–27)			
**SES**	**Low**	8.2(6.24)	7(0–32)	-0.19	0.0002 *	<0.0001 *
	**Moderate**	5.23(5.34)	4(0–30)			
	**High**	5.42(5.28)	4(0–28)			
**Level of education**	**Low**	9.48(6.93)	9(0–32)	-0.21	<0.0001 *	<0.0001 *
	**Moderate**	6.18(5.44)	5(0-29)			
	**High**	5.3(5.26)	4(0–30)			
**Brushing frequency**	**No brushing**	8.95(6.71)	8(0-32)	-0.3	<0.0001 *	<0.0001 *
	**Infrequent**	5.84(4.18)	6(0–19)			
	**once daily**	6.06(5.31)	5(0–30)			
	**Twice daily**	5.04(5.76)	4(0–29)			
	**Three times**	3.64(3.69)	2(0–16)			
	**Other**	6.5(5.97)	6(0–14)			
**Dietary Habits**						
** -Bread**	**≤ 2 times/week**	6.69(5.23)	6(0–16)	0.08	0.1414	0.9858
	**3-6 times/week**	2.73(2.87)	2(0–9)			
	**1-6 times/day**	6.22(5.78)	5(0–32)			
** -Other carbohydrates**	**≤ 2 times/week**	7.05(6.42)	6(0-30)	-0.03	0.5668	0.2368
	**3-6 times/week**	5.36(5.84)	4(0–32)			
	**1-6 times/day**	5.97(5.47)	5(0–28)			
** -Eggs**	**≤ 2 times/week**	6.17(5.5)	5(0–32)	-0.04	0.4302	0.2004
	**3-6 times/week**	4.92(4.35)	4(0–17)			
	**1-6 times/day**	7.69(8.06)	4(0–29)			
** -Fruits/Vegetables**	**≤ 2 times/week**	6.29(5.25)	5(0–29)	-0.04	0.4687	0.6645
	**3-6 times/week**	5.56(4.54)	5(0–2 1)			
	**1-6 times/day**	6.16(6.08)	5(0–32)			
** -Milk**	**≤ 2 times/week**	6.43(5.75)	5(0–32)	-0.08	0.1498	0.287
	**3-6 times/week**	6.28(5.68)	6(0–30)			
	**1-6 times/day**	5.63(5.65)	5(0–28)			
** -Milk products**	**≤ 2 times/week**	6.66(5.95)	5(0–32)	-0.06	0.2221	0.3557
	**3-6 times/week**	5.78(5.85)	4(0–30)			
	**1-6 times/day**	5.86(5.55)	5(0–29)			
** -Grains**	**≤ 2 times/week**	6.33(5.9)	5(0–29)	-0.04	0.3965	0.5547
	**3-6 times/week**	6.41(5.8)	5(0–30)			
	**1-6 times/day**	5.74(5.48)	4(0–32)			
** -Sugar in drinks**	**≤ 2 times/week**	6.34(4.76)	6(0–26)	-0.07	0.1756	0.3963
	**3-6 times/week**	5.67(3.77)	5.5(1–14)			
	**1-6 times/day**	6.05(5.97)	5(0–32)			
** -Sugar not in drinks**	**≤ 2 times/week**	6.51(6.06)	5(0–32)	-0.09	0.0928	0.2055
	**3-6 times/week**	5.38(2.78)	4.5(1–9)			
	**1-6 times/day**	5.13(4.95)	4(0–30)			
** -Jam, Molasses and Honey**	**≤ 2 times/week**	6.09(5.62)	5(0–32)	-0.02	0.7405	0.9328
	**3-6 times/week**	5.97(5.85)	5(0–30)			
	**1-6 times/day**	6.14(6.04)	4(0–29)			
** -Candies**	**≤ 2 times/week**	6.8(6.17)	5(0–32)	-0.18	0.0008 *	0.0036 *
	**3-6 times/week**	4.28(3.25)	4(0–13)			
	**1-6 times/day**	4.58(4.36)	3(0–22)			
** -Crackers**	**≤ 2 times/week**	6.85(6.4)	5(0–32)	-0.11	0.0374	0.0839
	**3-6 times/week**	4.78(4.01)	4.5(0–16)			
	**1-6 times/day**	5.26(4.69)	4(0–27)			
** -Junk food**	**≤ 2 times/week**	6.42(6.2)	5(0–32)	-0.05	0.3459	0.4277
	**3-6 times/week**	5.84(3.67)	6(0–12)			
	**1-6 times/day**	5.31(4.67)	4(0–20)			
** -Chocolate**	**≤ 2 times/week**	6.51(5.98)	5(0–32)	-0.12	0.0227 *	0.075
	**3-6 times/week**	5.06(4.27)	4(0–16)			
	**1-6 times/day**	4.94(4.94)	4(0–26)			
** -Soda drinks**	**≤ 2 times/week**	6.14(5.9)	5(0–32)	-0.02	0.733	0.2116
	**3-6 times/week**	6.8(4.59)	6(0–18)			
	**1-6 times/day**	5.77(5.65)	4(0–32)			
** -Juices**	**≤ 2 times/week**	6.78(5.54)	5(0–28)	-0.21	<0.0001 *	0.0002 *
	**3-6 times/week**	5.3(7.63)	4(0–32)			
	**1-6 times/day**	4.85(5.21)	4(0–29)			
** -Citric juices**	**≤ 2 times/week**	6.25(5.84)	5(0–32)	-0.05	0.3574	0.6451
	**3-6 times/week**	5.84(5.49)	4(0–25)			
	**1-6 times/day**	5.47(5.13)	4(0–27)			
** -Caffeinated drinks**	**≤ 2 times/week**	5.02(5.4)	4(0–26)	0.06	0.2382	0.1643
	**3-6 times/week**	6.21(3.09)	5.5(2–14)			
	**1-6 times/day**	6.25(5.83)	5(0–32)			

The correlation coefficient, rho, ranges from -1 to +1. Where 1= perfect positive correlation, 0=no correlation, -1 = perfect negative (inverse) correlation. *Statistical significance at p-value ≤ *0.05.*

Males had a higher mean DMFT teeth than females (6.5±5.47). The difference in medians was statistically insignificant (p≥0.05) and there was no correlation between gender and DMFT (Spearman’s rho= -0.09, p≥0.05).

Adults with low SES, low education level and those who don’t brush their teeth had the highest mean DMFT numbers (8.2±6.24, 9.48±6.93 and 8.95±6.71, respectively). The differences in medians were statistically significant (p<0.0001). SES, education level and brushing frequency were inversely correlated with DMFT (Spearman’s rho=-0.19, -0.21 and -0.3; p=0.0002, <0.0001 and <0.0001, respectively).

The highest means of DMFT, were recorded in adults who consume eggs, jam and caffeinated drinks 1–6 times per day (7.69±8.06, 6.14±6.04 and 6.25±5.83, respectively) and those who consume grains and soft drinks 3–6 times per week (6.41±5.8 and 6.8±4.59, respectively), as well as those who consume bread, other carbohydrates, fruits/vegetables, milk, milk products, sugar in drinks, sugar not in drinks, candies, crackers, junk food, chocolate, juices and citric juices two or fewer times a week (6.69±5.23, 7.05±6.42, 6.29±5.25, 6.43±5.75, 6.66±5.95, 6.34±4.76, 6.51±6.06, 6.8±6.17, 6.85±6.4, 6.42±6.2, 6.51±5.98, 6.78±5.54 and 6.25±5.84, respectively). The differences in medians for DMFT in all dietary elements were statistically insignificant except for candies and juices. Consumption of candies, chocolate and juices were inversely correlated with DMFT.

## Discussion

To our knowledge, the present study is the first to clarify the prevalence of dental caries and treatment needs among Egyptian adults and their correlation with different risk factors. Egyptian adults proved to be at greater risk of developing caries, with a total prevalence of 86.63%; as compared to children and adolescence, for whom a value of 74% was recorded ^16^. The recorded DMFT in the present investigation is lower than that recorded in North-West Russia (96%) ^19^, higher than that recorded in Turkey (62%) ^20^ and much higher than the prevalence of caries in England (31%) ^21^.

In the current work, DMFT with its components M and F showed a significant increase with age (P<0.0001), this is in agreement with previous studies carried out in Australia and China ^22, 23^, but contrary to a study performed in Turkey, which reported a decrease in dental caries with age ^20^.

No significant correlation between gender and caries has been reported in the current investigation. This is in contrast with other studies, which reported higher caries indices in females ^23, 24^, while another study reported a significant increase in decayed teeth in males ^25^.

Both dental caries and BMI are diet-related health measures. The incidence of caries and obesity has raised in the last two decades due to changes in lifestyle and diet ^26, 27^. The reported significant positive correlation between BMI, DMFT and missing teeth in the current study is in accordance with findings by Sheiham *et al*., who showed that British people that had less than 20 teeth were more likely to be obese ^28^; however, a study performed on Korean adults revealed an inverse correlation ^29^.

SES and education level are thought to be strongly associated with dental caries rate. In the present work, there were inverse correlations between socioeconomic, education levels and number of decayed, missing teeth and DMFT, while a positive correlation with filled teeth. These findings reinforce those of previous studies ^30– 33^ and are partially in agreement with those of Ceylan *et al*. ^34^, who found that the mean number of filled teeth was strongly correlated with income level, while DMFT was not correlated with income and education level.

Regarding oral hygiene, DMFT and the mean number of missing and decayed teeth were significantly higher in adults who don’t brush their teeth (8.95±6.71, 3.34±5.57, 4.26±5.76, respectively). These results are similar to studies conducted by Fukuda *et al*. ^35^ and Levin *et al.* ^36^, who reported that regular tooth brushing improved the oral health.

Dietary habits are important contributors to the heath or disease of a population. Alteration in dietary habits, like increased consumption of refined sugars, soft drinks and fast food, cause caries as well as obesity ^37^. In the present study, it was found that adults who consumed bread, grains, crackers, junk food, chocolate, soft drinks and caffeinated drinks 1–6 times per day had the highest mean number of decayed teeth, with a significant positive correlation observed with grain, junk food and soft drink consumption. This is in accordance with results obtained by Jones *et al*., who reported a significant correlation between soft drink consumption and DMFT index ^38^.

Despite evidence from previous studies, which revealed a correlation between caries incidence and sugar intake in children ^39, 40^ and in adults ^34^, the present investigation reported weak correlations or even negative correlations between cariogenic food and the number of missing, filled teeth and DMFT, which are in accordance with other studies ^41– 43^. Consumption of sweetened foods and drinks between meals usually leads to the development of caries in children, while is associated with obesity in adults ^44^. An indication of an answer to the controversial question “how obesity causes dental caries?” could be found in a recent cross-sectional study carried out on adolescences. The authors investigated the relationship between obesity and bite force. They inferred that the decreased bite force reported in obese males and females might result in a preference for soft food stuffs and a reduction in chewing, which in turn might cause caries. In contrast, individuals with normal body weight, have increased bite force and choose harder foodstuffs ^45^.

On the contrary, some diets may favour remineralization when their content is high in calcium, phosphate and protein ^46^. This is confirmed by the significant negative correlation between milk consumption and the number of decayed teeth reported in the current study.

A limitation of this cross-sectional study is that the collected dietary information covered only a few short periods in time, which may not be an accurate representation of an individual's actual lifetime dietary habits. This could explain the lack of significant differences between some caries indices and cariogenic dietary elements. The DMFT index is the most widely used index worldwide, but it has some limitations. The F or M component might not only display teeth that were previously decayed and could include other conditions not related to dental caries ^47^.

From this study, it could be concluded that age, BMI, SES, education level and brushing frequency are risk factors significantly associated with caries prevalence amongst Egyptian adults.

## Data availability

### Underlying data

Raw data, including all answers to the questionnaire and data on caries incidence amongst the sampled population, are available on figshare. DOI: https://doi.org/10.6084/m9.figshare.7609832.v1 ^18^.

### Extended data

A copy of the study questionnaire is available on figshare. DOI: https://doi.org/10.6084/m9.figshare.7609733.v1 ^9^.

Data are available under the terms of the Creative Commons Zero "No rights reserved" data waiver (CC0 1.0 Public domain dedication).
