# Supplementary material for: The potential impact of age, gender, body mass index, socioeconomic status and dietary habits on the prevalence of dental caries among Egyptian adults: a cross-sectional study
Source: F1000Res. 2019 Mar 1;8:243. [Version 1] doi: 10.12688/f1000research.17892.1 (PMC6426081; doi:10.12688/f1000research.17892.1)
Supplement: Peer review history

# Peer review: Reviewer response for version 1

**DOI:** 10.5256/f1000research.19568.r45144

**Published:** 2019-05-10

**Authors:** Wafa El-Badrawy

**Affiliations:** 1 Faculty of Dentistry, University of Toronto, Toronto, ON, Canada

The subject of the research is new for the area and it will be of value to the profession. The study is well designed, the manuscript is well written and edited however from the research point of view the study is lacking objectives and hypotheses. The methods are clear and well explained. In the result section: the number of tables is excessive which makes it slightly complicated for the reader to follow. Conclusion are clear and well written.

I have read this submission. I believe that I have an appropriate level of expertise to confirm that it is of an acceptable scientific standard.

# Peer review: Reviewer response for version 1

**DOI:** 10.5256/f1000research.19568.r45143

**Published:** 2019-03-15

**Authors:** Tahra Elobeid

**Affiliations:** 1 Human Nutrition Department, College of Health Sciences, Qatar University, Doha, Qatar

With the high prevalence of obesity and Non communicable diseases (NCDs), it is important to investigate how it affects the other underlying problems associated with it. This study provides very important findings as research in this area is still virgin in the region. The change in the food habits and nutrition transition further complicates this problem. Oral health and NCDs risk factors is established in several studies however the correlations were not significant. The association between dental health and obesity has not been studied in the region although there has been a significant change in the food habits and socioeconomic levels of the different populations. This will further increase the burden on public health system.

I have read this submission. I believe that I have an appropriate level of expertise to confirm that it is of an acceptable scientific standard.

# Peer review: Reviewer response for version 1

**DOI:** 10.5256/f1000research.19568.r45145

**Published:** 2019-03-12

**Authors:** Mohammed E. Grawish

**Affiliations:** 1 Department of Oral Biology, Faculty of Dentistry, Mansoura University, Mansoura, Egypt

The paper by Abbass et al., evaluated clearly and accurately the potential impact of age, gender, body mass index, socioeconomic status and dietary habits on the prevalence of dental caries among Egyptian adults. The title and abstract are appropriate for the content of the text. The cross-sectional study design is appropriate and the work is technically sound. Sufficient details of methods and analyses were provided and it has an external validity as well as internal one and thus it can be reproduced. Non parametric statistical tests were performed as the data are categorical in type. The data obtained are interpreted appropriately and the results are valid. All the source data underlying the results are available and thus full reproducibility can be attained. The conclusions drawn are adequately supported by the results. In addition, the paper is well written and precisely formatted.

I have read this submission. I believe that I have an appropriate level of expertise to confirm that it is of an acceptable scientific standard.
